# Electromagnetic tracking in image‐guided laparoscopic surgery: Comparison with optical tracking and feasibility study of a combined laparoscope and laparoscopic ultrasound system

**DOI:** 10.1002/mp.13210

**Published:** 2018-10-19

**Authors:** Guofang Xiao, Ester Bonmati, Stephen Thompson, Joe Evans, John Hipwell, Daniil Nikitichev, Kurinchi Gurusamy, Sébastien Ourselin, David J. Hawkes, Brian Davidson, Matthew J. Clarkson

**Affiliations:** ^1^ Wellcome/EPSRC Center for Interventional and Surgical Sciences University College London London UK; ^2^ Center for Medical Image Computing University College London London UK; ^3^ Department of Medical Physics and Biomedical Engineering University College London London UK; ^4^ Division of Surgery and Interventional Science University College London London UK

**Keywords:** calibration, electromagnetic tracking, image‐guided surgery, laparoscopic surgery, optical tracking

## Abstract

**Purpose:**

In image‐guided laparoscopy, optical tracking is commonly employed, but electromagnetic (EM) systems have been proposed in the literature. In this paper, we provide a thorough comparison of EM and optical tracking systems for use in image‐guided laparoscopic surgery and a feasibility study of a combined, EM‐tracked laparoscope and laparoscopic ultrasound (LUS) image guidance system.

**Methods:**

We first assess the tracking accuracy of a laparoscope with two optical trackers tracking retroreflective markers mounted on the shaft and an EM tracker with the sensor embedded at the proximal end, using a standard evaluation plate. We then use a stylus to test the precision of position measurement and accuracy of distance measurement of the trackers. Finally, we assess the accuracy of an image guidance system comprised of an EM‐tracked laparoscope and an EM‐tracked LUS probe.

**Results:**

In the experiment using a standard evaluation plate, the two optical trackers show less jitter in position and orientation measurement than the EM tracker. Also, the optical trackers demonstrate better consistency of orientation measurement within the test volume. However, their accuracy of measuring relative positions decreases significantly with longer distances whereas the EM tracker's performance is stable; at 50 mm distance, the RMS errors for the two optical trackers are 0.210 and 0.233 mm, respectively, and it is 0.214 mm for the EM tracker; at 250 mm distance, the RMS errors for the two optical trackers become 1.031 and 1.178 mm, respectively, while it is 0.367 mm for the EM tracker. In the experiment using the stylus, the two optical trackers have RMS errors of 1.278 and 1.555 mm in localizing the stylus tip, and it is 1.117 mm for the EM tracker. Our prototype of a combined, EM‐tracked laparoscope and LUS system using representative calibration methods showed a RMS point localization error of 3.0 mm for the laparoscope and 1.3 mm for the LUS probe, the lager error of the former being predominantly due to the triangulation error when using a narrow‐baseline stereo laparoscope.

**Conclusions:**

The errors incurred by optical trackers, due to the lever‐arm effect and variation in tracking accuracy in the depth direction, would make EM‐tracked solutions preferable if the EM sensor is placed at the proximal end of the laparoscope.

## 1 Introduction

Laparoscopic surgery has significant advantages over open surgery due to less patient trauma and faster recovery times, yet it can be difficult to perform due to the restricted field of view and lack of haptic feedback. Image guidance, and specifically augmented reality, has been proposed as a way to alleviate this problem and reduce the risk of complication.^1^ Most guidance systems use optical tracking.^2^, ^3^, ^4^, ^5^ When using optical tracking, to ensure a free line‐of‐sight, markers must be placed on the distal end of the laparoscope, far from the optical center of its camera, thereby creating a lever‐arm effect, reducing system accuracy. Our previous work has shown that an EM sensor placed at the proximal end of the laparoscope should provide higher system accuracy.^6^ In this study, we performed a detailed evaluation of tracking precision and accuracy, comparing optical and EM tracking, specifically for laparoscopy. We also carried out a feasibility study of a combined, EM‐tracked laparoscope and laparoscopic ultrasound (LUS) image guidance system.

### 1.A. Background

In the field of image‐guided surgery, optical tracking is widely regarded as being accurate and reliable.^7^ However, users of tracking devices often rely on a single measure of accuracy published on a manufacturer's website as a summary statistic. This summary statistic is often based on measurements from a single marker or sensor, tracked under laboratory conditions. For optical trackers, the accuracy can vary substantially throughout the tracking volume and may suffer from systematic errors.^8^ When designing an optically tracked tool, individual markers are combined into a marker set, which has a defined and accurately manufactured configuration. It has been shown that the ability to accurately locate a target, such as an instrument tip, depends on the error distribution in detecting each marker, the number and spatial distribution of the markers, and the distance of the target from each principal axis of the marker set.^9^ Consequently, the system accuracy can be very different from the manufacturer's quoted accuracy and an application‐specific accuracy assessment must be performed.^8^ The combination of size and shape of the marker set, along with the distance from the laparoscope camera, is particularly problematic for optically tracking laparoscopes where the tip must be placed within the abdominal cavity and may be some 30–50 cm from the centroid of the markers, and the size of the marker set cannot be increased too much as it must not impinge on the trocar or hamper the surgeon's operation.

Electromagnetic‐tracking benefits from no line‐of‐sight issues and sensors can be placed at the proximal end of the instruments. Furthermore, where flexible instruments are required,^10^, ^11^ EM tracking becomes the natural choice.^12^ Unfortunately, the accuracy and reliability can suffer from magnetic field distortions due to the presence of electronic devices or ferromagnetic objects nearby. A comprehensive review of EM tracking in medicine is provided by Franz et al.^12^ Efforts have been made to develop standardized protocols for assessment of static errors^13^, ^14^ and dynamic errors^15^, ^16^ of EM trackers. As new trackers become available, they can be systematically evaluated.^10^, ^17^, ^18^, ^19^, ^20^ Currently, no such standardized evaluation has been performed for laparoscopy and certainly not for an EM‐tracked laparoscope.

Both optical and EM‐tracking systems exist for laparoscopy.^21^, ^22^ The group at Children's National Medical Center in Washington moved from an optically tracked to an EM‐tracked system^21^, ^23^ to reduce line‐of‐sight issues and ultimately to combine a laparoscope with a flexible LUS probe which necessitates EM tracking. However, in that work,^23^ the EM sensor was placed on the distal end of the laparoscope, which must compound the poorer intrinsic level of accuracy of the EM tracker with the lever‐arm effect, leading to suboptimal localization of the camera. That said, no commercially available solution with an EM‐tracked laparoscope and an EM‐tracked LUS exists, so this is a reasonable interim solution, and a good developmental step.

### 1.B. Theory

For a tracked laparoscope or LUS probe, system accuracy is affected by the tracking error of the marker set or sensor attached to the device. In laparoscopy, there are two sources of error with optical tracking that are often underestimated, which are described below and demonstrated via experiments.

#### 1.B.1. Lever‐arm effect error

**Figure 1:**
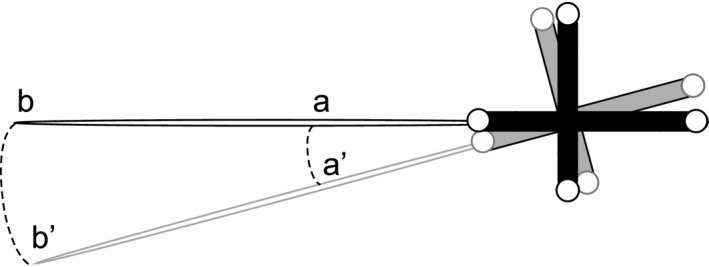
Illustration of the lever‐arm effect error, using an optical marker set as an example of the tracked frame. Error in orientation measurements causes more misplacement for Point *b* which is further away from the tracked frame than for Point *a*.

In this paper, we use the term *tracked frame* to refer to either the marker set tracked by an optical tracker or the EM sensor tracked by the EM tracker. For any tracking system, error exists in both the position and orientation measurement of the tracked frame. Error in position measurement does not have a varying effect on the localization of the target point in relation to its distance from the origin of the tracked frame. However, for orientation measurement, the error causes more misplacement of the target point when it is further away from the tracked frame. This is usually called the “lever‐arm effect”. Figure 1 illustrates this effect, using an optical marker set as an example of the tracked frame. For EM trackers, error in orientation measurement is usually larger than for optical trackers; however, when EM sensors are placed close to the instrument tip to minimize the lever‐arm effect, they can perform better in terms of precision and accuracy than the optical trackers, as will be shown through our experiments.

#### 1.B.2. Depth reconstruction error of optical trackers

**Figure 2:**
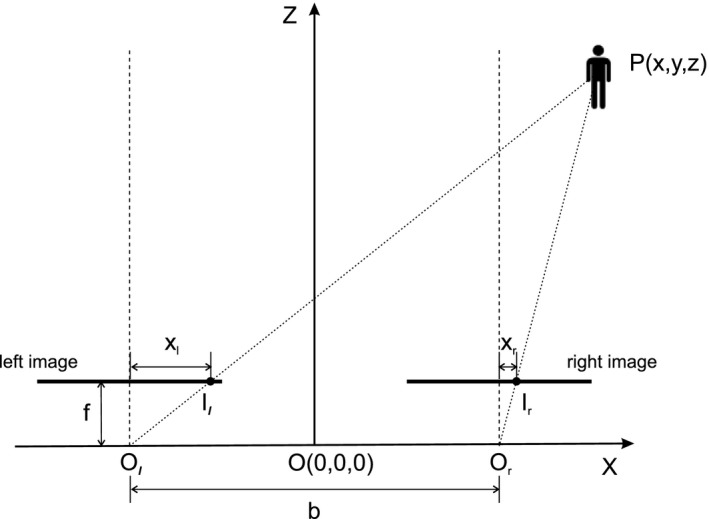
Principle of stereo camera vision.

Optical trackers use stereo cameras to image the markers, detect and match the markers in the left and right images, and then use stereo triangulation to calculate the three‐dimensional (3‐D) coordinates of the markers. A canonical stereo camera system is illustrated in Fig. 2. The two cameras are mounted such that their optical axes are coplanar and perpendicular to the line connecting their optical centers *O*_*l*_ and *O*_*r*_ which is called the baseline and its length denoted as *b*. In practice, the optical axes of the two cameras are pointing inward slightly instead of parallel, but this does not make a material difference to the analysis here. Let the middle point of the baseline be the origin *O* and the *Z* axis be perpendicular to the image planes. The image points *I*_*l*_ (*x*_*l*_ *, y*_*l*_) on the left image plane and *I*_*r*_ (*x*_*r*_ *, y*_*r*_) on the right plane are the projections of a point *P* (*x, y, z*) in 3‐D space. Let the two cameras have the same focal length *f*, which is the distance from the optical centers to the image plane for each camera. From the relationship between similar triangles, the depth of point *P* is:

(1) $$Z=\frac{\mathrm{bf}}{d}$$

where *d *= *x*_*l*_ *− x*_*r*_ and is referred to as the *disparity*. As can be seen that the disparity is inversely proportional to the depth. With a little calculus, we get

(2) $$\delta z=-\frac{z^{2}}{\mathrm{bf}}\delta d.$$

The above equation shows the relationship between the change in the disparity measured, *δd*, and the change caused in the depth reconstructed, *δz*. It indicates that for a certain amount of error in disparity measure, the error in the reconstructed depth increases quadratically with the distance from the camera. Optical trackers used in image‐guided surgery usually have a working distance of 700–3000 mm; hence, *z* is always big, which leaves little room for reduction in *δz*. From Eq. (2), we can also see that the accuracy of depth reconstruction will decrease with the increase in *b*. However, a larger *b* not only means a bulkier device but also more differences in the left and right images hence more difficulty in finding the correspondences. A larger *f* can also decrease depth reconstruction error but at the same time results in narrower field of view.

### 1.C. Contributions of this paper

In this paper, we test the counterintuitive theory that an EM‐based system can outperform an optically tracked system for laparoscopy. Specifically, we contribute the following:

- We measured the tracking accuracy of a laparoscope with two optical trackers and an embedded EM tracker and found that while optical trackers appear to have an intrinsically better tracking capability, they display significant errors in the depth direction, leading to worse accuracy in relative position measurement than the EM tracker.
- We subsequently found that with the sensor placed at the proximal end of the laparoscope, the EM tracker can provide more precise and more accurate measurements than the optical trackers which suffer from lever‐arm effect error.
- We evaluated the system‐wide accuracy of an EM‐tracked image guidance system incorporating a laparoscope and a LUS probe.

## 2 Materials and methods

In this study, three experiments were carried out. In Experiment 1 (Section 2.A) and Experiment 2 (Section 2.B), we test the performance of EM tracking with the EM sensor embedded inside the laparoscope and study how this compares to optical tracking. Three tracking systems were evaluated. They are an Atracsys (Atracsys, Puidoux, Switzerland) Fusion Track 500 optical tracker and an NDI (Northern Digital Inc., Waterloo, ON, Canada) Polaris Spectra optical tracker, along with an NDI Aurora V3 EM tracker with the NDI Tabletop Field Generator (TTFG). The optical trackers tracked the same NDI optical marker set (part 8700339), which has four reflective optical markers and was mounted on the shaft of a Storz (Karl Storz GmbH & Co. KG, Tuttlingen, Germany) Hopkins laparoscope (model 26038AA). The EM system tracked an Aurora six degree of freedom (DoF) Type 2 Flex Tube, 1.3 mm in diameter and 2 m long, with the sensor at the end. The EM sensor was passed down the working channel of the laparoscope and secured at the tip with a specially designed ferrule made of acetal. The Aurora TTFG has a working volume with an approximately elliptical cross section of 600 and 420 mm in axial diameter, located between 120 and 600 mm above the physical board. In our experiments, the TTFG was placed on a surgical bed, while the Atracsys and NDI Spectra cameras were mounted on a stand and positioned in a way that the space above the TTFG was within the working volumes of both optical tracking systems. In Experiment 3 (Section 2.C), we study the feasibility of a combined, EM‐tracked laparoscope and LUS system.

All the experiments were carried out in a normal laboratory environment, with devices including an ultrasound machine, a laparoscopic stack, and a desktop computer around the surgical bed, similar to a standard clinical setup. Data acquisition was carried out using the NifTK software platform,^24^ which recorded data from all three trackers in parallel. The Atracsys optical tracker operates at 335 Hz, the NDI Spectra optical tracker at 60 Hz, and the NDI Aurora EM tracker at 40 Hz.

### 2.A. Static accuracy assessment

**Figure 3:**
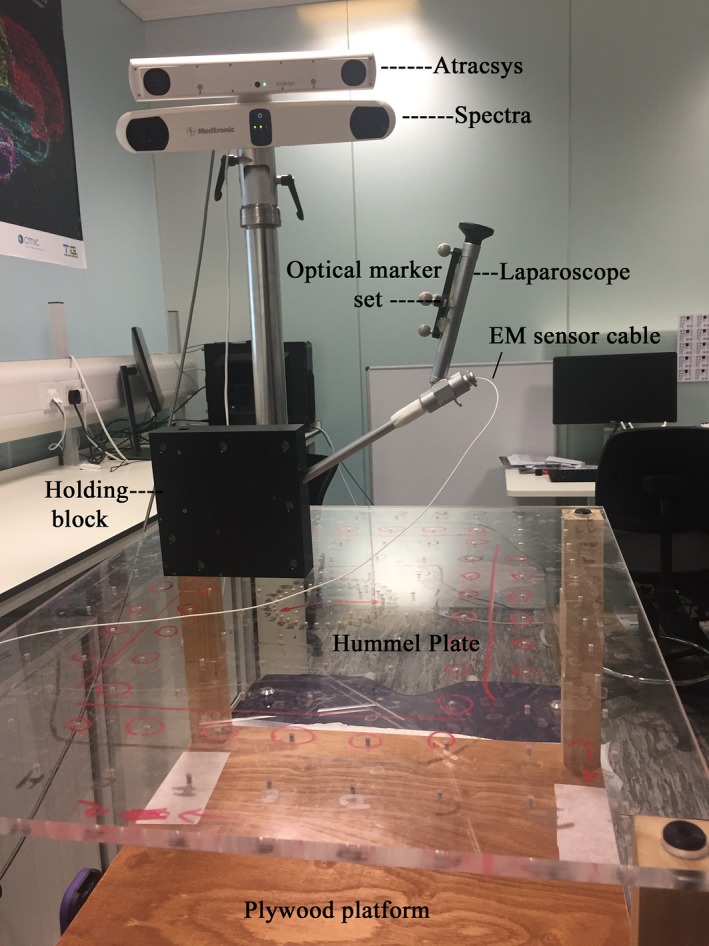
Experimental setup for static measurement accuracy assessment using the Hummel Plate. [Color figure can be viewed at http://www.wileyonlinelibrary.com]

The physical setup of this experiment is shown in Fig. 3. A methacrylate plate was fabricated according to the design of Hummel et al.^13^ and used as a ground truth. The 650 *× *550 *× *12 mm plate contains a 12 *× *10 regular grid of 3 mm diameter holes spaced 50 mm apart in each direction, with a precision of 10 μm at a temperature of 20°. A modular marine plywood platform was secured to the TTFG, which allows the plate to be easily positioned at three vertical levels (120, 220, and 320 mm) above the base to enable the assessment of accuracy in the vertical direction.

An acetal block was made to rigidly hold the laparoscope. The laparoscope was inserted into a tunnel inside the block and secured in place with a grub screw. A pair of pegs on the underside of the block, separated by 50 mm, enables the block to be securely attached to the grid of holes in various positions. The block can be positioned on the grid in two directions: along the row direction (as in Fig. 3) or along the column direction of the grid, while keeping the optical markers visible to the cameras. For the first (row‐wise) direction, the optical marker set is fairly oblique to the optical tracker, as shown in Fig. 3. For the second (column‐wise) direction, the optical marker set is facing straight at the cameras.

In this study, the central 8 *× *6 holes of the grid were used for assessment, as these holes were within the working volumes of all three trackers at all three levels and for both directions.

#### 2.A.1. Jitter

Continuous measurements from a static sensor contain random error, commonly referred to as *jitter*. At each grid point, 10 s of a continuous stream of position and orientation measurements were recorded from all three trackers. The root‐mean‐square (RMS) of the Euclidean distance between each measured position and the mean position of all measurements was calculated and called *position jitter*. The orientation measurements recorded were in unit quaternion form. For ease of comprehension, each quaternion was converted to three Euler angles, and the RMS error of the angles was computed in the same way as the position coordinates and used as *orientation jitter*.

#### 2.A.2. Orientation error

When the holding block is moved along one direction (the row direction or the column direction) on the grid, its orientation in each tracker coordinate system remains unchanged. Therefore, the variation in orientation measurements across the grid indicates the orientation measurement accuracy of the tracking system. For each tracker, at each grid point, the mean orientation was obtained by computing the mean of all orientation measurements for each Euler angle (thereby eliminating orientation jitter). The standard deviation (SD) of the mean orientation across the 8 *× *6 grid and of all three levels was calculated, averaged over three Euler angles, and used as orientation error. We decided not to use the ring feature of the Hummel Plate for the evaluation of orientation measurement accuracy and this is discussed in Section 4.

#### 2.A.3. Relative position error

For each tracker, at each grid point, the mean position was calculated by averaging all position measurements (thereby eliminating position jitter). Relative position errors were determined by comparing the Euclidean distances between the grid points calculated using their mean positions, with their known physical distances on the grid. RMS errors for distances of 50, 150, and 250 mm in two directions were computed.

### 2.B. Stylus‐based accuracy assessment

**Figure 4:**
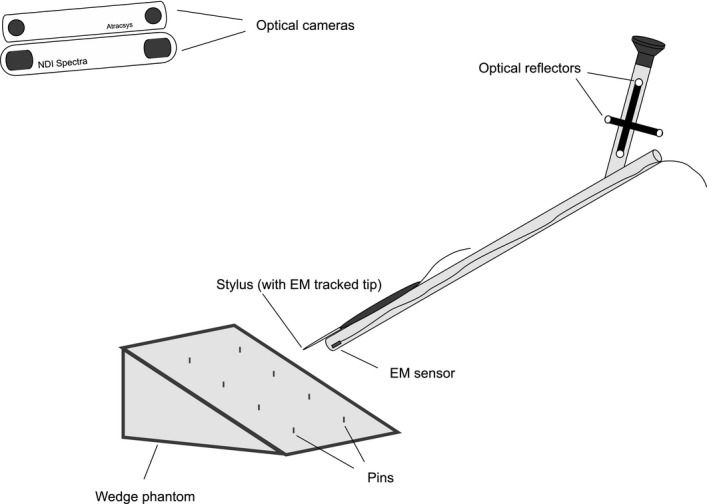
Experimental setup for stylus measurement accuracy assessment.

In the second part of our study, we investigate the effects of lever‐arm length on the precision and accuracy of optical and EM trackers. A stylus was attached to the laparoscope used in the first experiment. The stylus is an NDI Aurora 6 DoF digitizing probe (part number 610065), with a rigid, straight metal tip. It has a built‐in EM sensor, and the position and orientation of the tip are tracked. It was connected to the TTFG together with the EM sensor in the laparoscope, so they used the same reference coordinate system and their measurements are directly comparable. The stylus was securely taped to the laparoscope body, with the tip about 3 cm away from the proximal end of the laparoscope. We used the stylus to take measurements of a wedge phantom^25^ (Fig. 4). The wedge phantom is made of a tough plastic and has eight stainless steel pins, 0.5 mm in diameter and protruding by 1 mm perpendicular to the inclined surface. The pins are arranged as a 4 *× *2 grid, with 25 mm spacing. The stylus was pointed at the tip of each pin and held stationary. Three seconds of continuous measurements from the three trackers were recorded. This was done from four different directions to coarsely sample the operative orientation range of the optical cameras.

Pivot calibration^26^ was performed to determine the offset of the tip of the stylus from the origin of the tracked frame for each of the three trackers as used in the first experiment, that is, the two optical trackers using the same optical marker set and the EM tracker with the sensor fixed at the tip inside the laparoscope. For the Aurora stylus, (0*,* 0*,* 0) was used as the offset. For each pin, the following transformation was applied for every measurement:

(3) $$p=R\cdot v_{\mathrm{offset}}+t,$$

where **v**_offset_ is the offset vector solved by pivot calibration, **R** and **t** are the rotation matrix and the translation vector representing the orientation and position measurements from the tracker, respectively, and **p** is the position of the pin in the tracker's coordinate system.

#### 2.B.1. Precision of position measurement

For each tracker, 360 samples were randomly selected from all the recordings of each pin (hence, each sample is a measurement taken from a random one of the four directions aforementioned). All samples were transformed according to Eq. (3) into each tracker's coordinate system, resulting in a point cloud for each of the eight pins and for each tracker. We define the “tightness” of each point cloud as the RMS of the Euclidean distance between each point in the given point cloud and the mean position of all points in that point cloud. The “tightness” is used to indicate the precision of position measurement of the tracker.

#### 2.B.2. Accuracy of distance measurement

The Euclidean distances between pairs of pins were computed, using their positions measured by the trackers and transformed according to Eq. (3), and compared with their known physical distances, including diagonal combinations, that is, distances of 25.00, 35.36, 50.00, 55.90, 75.00, and 79.06 mm. For each pair of pins under consideration, a random sample was chosen from all the recordings of each pin, for each tracker. The RMS error of all the measured distances was computed. This was done 360 times.

### 2.C. Accuracy assessment of an EM‐tracked system

**Figure 5:**
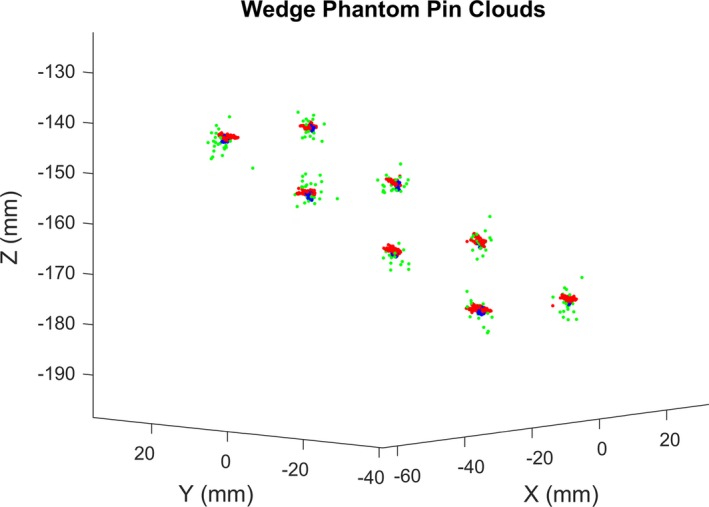
Point clouds from the measurements of the eight pins of the wedge phantom by the EM stylus (blue), EM‐tracked laparoscope (green), and LUS (red).

In the third part of our study, we evaluate the overall accuracy of an image‐guided laparoscopic surgery system. An Aurora 6 Dof EM sensor was fixed and sealed to the exterior surface and at the proximal end of a Viking 3DHD (CONMED Corporation, Utica, NY, USA) stereo laparoscope. Another such sensor was fixed and sealed next to the exterior surface of the transducer of a Vermon LAP7 (Vermon S.A., Tours, France) laparoscopic ultrasound probe. The same stylus employed in Experiment 2 (Section 2.B) was used to measure the position of the tip of each pin of the wedge phantom, from eight approximately evenly spaced directions, with 3 s of continuous measurements recorded for each direction. For each pin, a point cloud was formed from all the position measurements (Fig. 5, points in blue). The average point cloud tightness over eight pins and RMS error of measurements of all possible distances between any two pins and from all combinations of two position measurements were calculated.

#### 2.C.1. EM‐tracked laparoscope

Both video channels of the stereo laparoscope were calibrated. Intrinsic calibration was performed using Zhang's method,^27^ implemented in OpenCV.^28^ The stereo separation was also determined using OpenCV's stereo camera calibration routines. The position of the left lens relative to the EM tracker (hand‐eye calibration) was found using an initial linear method^29^ followed by nonlinear optimization using the Levenberg–Marquardt method to minimize the 2‐D projection errors.

Each of the eight pins on the wedge phantom was imaged with a 30 s (22.5 frames per second per channel) video acquisition. During the acquisition, the laparoscope was slowly swept through an arc of approximately 180°, keeping the pin approximately in the image center. After acquisition, the pin heads were manually located in 12 evenly spread stereo image pairs, allowing the pin position relative to the left lens to be triangulated.

The pin position relative to the left lens was then transformed to world coordinates using the EM‐tracking data and the hand‐eye calibration. The results are presented as a point cloud for each pin (Fig. 5, points in green) that can be directly compared to the pin positions measured with the Aurora stylus as well as those reconstructed from the LUS scans.

#### 2.C.2. EM‐tracked laparoscopic ultrasound (LUS)

**Figure 6:**
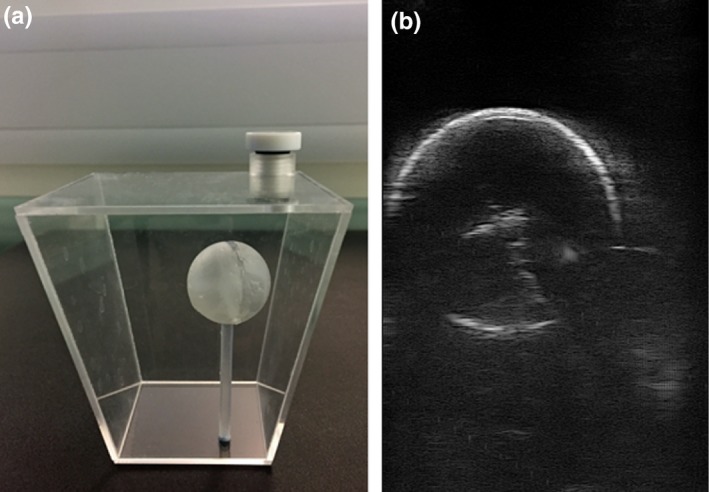
The ball‐and‐cross calibration phantom (a) and an ultrasound scan of the ball passing its center (b). [Color figure can be viewed at http://www.wileyonlinelibrary.com]

Hand‐eye calibration, which determines the transformation from the coordinate system of the 2‐D ultrasound scans to the coordinate system of the EM sensor being tracked, was performed for the EM‐tracked LUS probe using a “ball‐and‐cross” calibration phantom.^30^ The phantom is a 3‐D‐printed hollow ball (with holes on the surface to let water in) of 25 mm diameter and 1 mm thickness, placed inside a small water tank [Fig. 6(a)]. Inside the ball is a 3‐D‐printed cross with three lines intersecting at the center of the ball. The calibration is essentially a single‐point calibration, which is known for its good performance,^31^ with the single point being the center of the cross. The purpose of using a ball is to automate feature detection: when we see the cross under ultrasound, we will also see a clear circle around it, which is a great circle of the ball as shown in Fig. 6(b). Instead of trying to locate the center of the cross directly, which is usually done manually as the cross is often not clear enough for automatic detection, we apply the Hough transform to detect the circle surrounding it. The center of the circle found is the center of the cross, as designed.

Forty‐nine scans of the ball like the one in Fig. 6(b), taking from different directions and distances, were used for calibration. After the center of the circle was detected in each scan, the Levenberg–Marquardt algorithm was employed to solve for the unknowns — the scaling factors and the rigid transformation from the B‐scan image coordinate system to the coordinate system of the EM sensor being tracked. After laparoscope videos of the pins were taken, water was put into the container in which the wedge phantom was fixed at the base. The EM‐tacked LUS probe was used to scan each of the eight pins from four random directions, with 3  s of data recorded for each direction. Images in which the pin is not seen clearly were discarded. Then, the position of the tip of the pin in each ultrasound B‐scan image was manually located. For each pin, the following transformation was applied for every manually located pin position to obtain its *x*,* y*, and *z* coordinates in the tracker's coordinate system:

(4) $$\left(\begin{array}{c} x\\ y\\ z \end{array}\right)=R_{t}\cdot \left(R_{c}\cdot \left(\begin{array}{c} s_{x}\cdot u\\ s_{y}\cdot v\\ 0 \end{array}\right)+t_{c}\right)+t_{t},$$

where *u* and *v* are the *x* and *y* coordinates of the pin in the B‐scan image plane (the *z* coordinate is always zero), respectively, *s*_*x*_ and *s*_*y*_ *,* **R**_*c*_ and **t**_*c*_ are the scaling factors, rotation matrix and translation vector solved by hand‐eye calibration, respectively, and **R**_*t*_ and **t**_*t*_ are the rotation matrix and translation vector representing the orientation and position measurements from the tracker, respectively. Similarly, a point cloud was formed for each pin (Fig. 5, points in red).

#### 2.C.3. System‐wide performance

Finally, for each pin of the wedge phantom, the mean of the position measurements from the Aurora stylus was calculated and used as the ground truth for its position. The RMS errors of all the pin positions deduced from the EM‐tracked laparoscope and the EM‐tracked LUS were calculated.

## 3 Results

### 3.A. Static accuracy assessment

#### 3.A.1. Jitter

Tables 1 and 2 show the mean and SD of the position and orientation jitter across the 8 *× *6 grid and of all three levels, respectively. We can see that the two optical trackers have less jitter than the EM tracker, in both position and orientation measurement. Also, the optical trackers had noticeably less orientation jitter in Direction 2, when the optical marker set being tracked was facing straight at the cameras, while the EM tracker's performance was similar for the two directions, as expected.

**Table 1:** Position jitter (mean *± *SD in mm) of Atracsys (optical), Spectra (optical), and Aurora (EM) in two directions

	Atracsys	Spectra	Aurora
Direction 1	0.020 *± *0.003	0.034 *± *0.009	0.096 *± *0.082
Direction 2	0.017 *± *0.004	0.028 *± *0.007	0.096 *± *0.080

**Table 2:** Orientation jitter (mean *± *SD in degrees) of Atracsys (optical), Spectra (optical), and Aurora (EM) in two directions

	Atracsys	Spectra	Aurora
Direction 1	0.060 *± *0.010	0.116 *± *0.029	0.467 *± *0.340
Direction 2	0.011 *± *0.002	0.028 *± *0.006	0.429 *± *0.349

It is worth mentioning that the data we recorded show that at point (2, 4) of the 8 *× *6 grid of Level 2, Direction 1, the Atracsys optical tracker had an anomalous reading involving 11 frames of data which caused a change in z coordinate of up to 10.3 mm and an orientation error of 8.2°, while the NDI Spectra which was tracking the same optical marker set at the same time did not show any abnormality. The results of the Atracsys shown here were calculated after the 11 frames of erroneous data were removed.

#### 3.A.2. Orientation error

The results are shown in Table 3. The optical trackers demonstrate better consistency of orientation measurement within the test volume, especially in Direction 2.

**Table 3:** Orientation error: standard deviation in orientation measurements (in degrees) of Atracsys (optical), Spectra (optical), and Aurora (EM) in two directions

	Atracsys	Spectra	Aurora
Direction 1	0.322	0.304	0.650
Direction 2	0.147	0.161	0.438

#### 3.A.3. Relative position error

The results are shown in Table 4. For both optical trackers, the accuracy in measuring relative positions decreases significantly with longer distances. This is rather unexpected and we give a possible explanation in Section 4 . The EM tracker's performance is stable in this test.

**Table 4:** Relative position error: RMS errors (in mm) of relative positions measured by Atracsys (optical), Spectra (optical), and Aurora (EM), moving the acetal block in two directions for distances of (a) 50 mm, (b) 150 mm, and (c) 250 mm

	Atracsys	Spectra	Aurora
(a) RMS errors for pairs 50 mm apart			
Direction 1	0.210	0.233	0.214
Direction 2	0.240	0.261	0.223
(b) RMS errors for pairs 150 mm apart			
Direction 1	0.620	0.700	0.303
Direction 2	0.713	0.786	0.347
(c) RMS errors for pairs 250 mm apart			
Direction 1	1.031	1.178	0.367
Direction 2	1.308	1.441	0.406

### 3.B. Stylus‐based accuracy assessment

The resulting assessment in this part shows a combination of pivot calibration accuracy and tracking accuracy, which are always associated in real applications. The accuracy of a given pivot calibration is itself affected by the lever‐arm effect and the accuracy of the tracking system used to perform the calibration. In practice, pivot calibration and the actual position and orientation tracking would be done using the same tracking system, so our method provides a fair comparison between the three tracking systems. The RMS residuals of pivot calibration are 0.317, 0.411, and 0.263 mm for the optical trackers Atracsys and Spectra and the Aurora EM sensor at the tip of the laparoscope, respectively.

#### 3.B.1. Precision of position measurement

**Figure 7:**
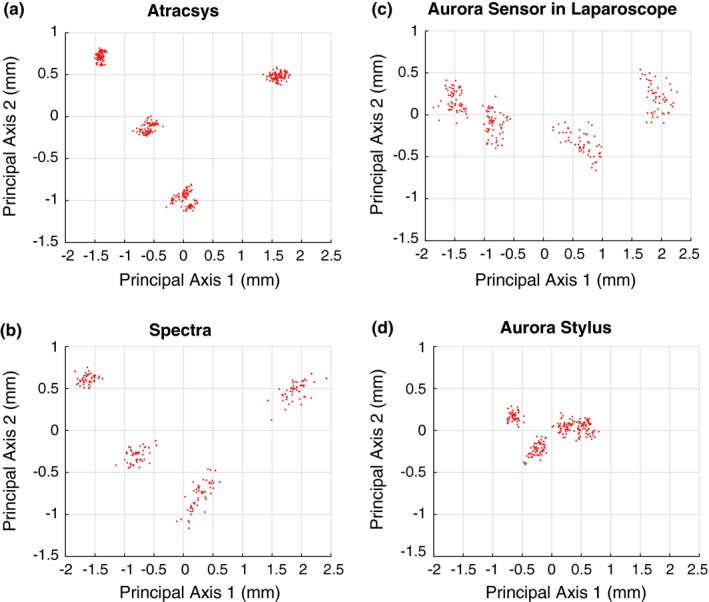
2‐D projections of point clouds of one pin of the wedge phantom from measurements of (a) Atracsys (optical), (b) Spectra (optical), (c) Aurora sensor (EM) in laparoscope, and (d) Aurora stylus (EM). [Color figure can be viewed at http://www.wileyonlinelibrary.com]

Figure 7 shows the point clouds of the data from a single pin of the wedge phantom from the measurements of Atracsys (a), Spectra (b), and Aurora, including the EM sensor inside the laparoscope (c) and the EM sensor in the stylus (d), respectively. Other seven pins produced similar looking plots. As the measurements from the three different tracking systems are in their own coordinate systems, it is difficult to choose one viewing point to look at and compare the 3‐D distribution of the point clouds. Hence, we performed principal component analysis (PCA) on each point cloud and projected it onto its first two principal axes. Recall that each pin was imaged from four different directions, resulting in four clusters per pin, per tracker. The tightness of each of the four clusters reflects the combination of jitter of the tracker, lever‐arm effect error, pivot calibration error as well as minuscule hand movements, while the distances between clusters are evidence of pivot calibration error and lever‐arm effect error. It can be seen clearly that for the optical tracker Atracsys (a), the four clusters are very tight on their own but are quite distant from each other, while the Aurora EM Stylus (d) has looser intracluster tightness, but the distances between clusters are smaller.

Table 5 gives the average point cloud tightness over eight pins of the wedge phantom for all the trackers. It shows that the Aurora sensor fixed at the proximal end of the laparoscope has better precision in localizing point target than the two optical trackers.

**Table 5:** Precision of position measurement (“tightness”): Mean RMS errors (in mm) of 360 randomly selected points for each of eight pins on the wedge phantom, of Atracsys (optical), Spectra (optical), Aurora sensor (EM) in laparoscope and Aurora stylus (EM)

	Atracsys	Spectra	Aurora	Aurora stylus
Tightness (mm)	1.278	1.555	1.117	0.523

#### 3.B.2. Accuracy of distance measurement

The mean RMS error over 360 trials of measuring all possible distances between any two pins is shown in Table 6 for each tracker. Again the Aurora results in superior accuracy compared to both optical trackers.

**Table 6:** Accuracy of distance measurement: RMS errors (in mm) over 360 trials of measuring all possible distances between any two pins, of Atracsys (optical), Spectra (optical), Aurora sensor (EM) in laparoscope and Aurora stylus (EM)

	Atracsys	Spectra	Aurora	Aurora stylus
Distance Err (mm)	1.218	1.309	1.004	0.498

### 3.C. Accuracy assessment of an EM‐tracked system

Figure 5 shows a plot of the points measured by the EM stylus (blue), the EM‐tracked laparoscope (green), and the EM‐tracked LUS probe (red). Table 7 shows the average point cloud tightness over eight pins of the wedge phantom and the RMS error of measurements of all possible distances between any two pins and from all combinations of two measurements, for each of the three trackers. Table 8 shows the RMS errors of pin positions reconstructed by both the laparoscope and LUS in relation to the EM stylus ground truth. The stylus illustrates the intrinsic EM‐tracking error. The errors for the Laparoscope include point picking error, stereo triangulation error, hand‐eye calibration error, and lever‐arm effect error. The errors for the LUS probe include point detection error, hand‐eye calibration error, and lever‐arm effect error. It can be seen that with a representative set of calibrations, the LUS probe would locate points accurate to 1.3 mm, while the laparoscope would result in accuracy around 3.0 mm. The point localization error of 3.0 mm of the EM‐tracked stereo laparoscope is significantly higher than the 1.117 mm position measurement precision of the EM sensor embedded at the tip of the laparoscope in Experiment 2 (Section 3.B.1). The larger error is likely to be predominantly due to the point triangulation error when using a narrow‐baseline (approximately 4.5 mm) stereo laparoscope. This magnitude of error is in line with our previous work on point triangulation using a stereo laparoscope.^22^ Given the evidence in Tables 5 and 6, we would expect optical trackers to be worse than EM trackers, largely due to the lever‐arm effect.

**Table 7:** Point cloud tightness and distance measurement accuracy: results from the EM stylus, EM‐tracked laparoscope, and EM‐tracked LUS

	EM stylus	Laparoscope	LUS
Tightness (mm)	0.524	2.864	1.017
Distance Err (mm)	0.485	2.403	1.050

**Table 8:** RMS errors of pin position reconstruction (in mm), using measurements from the EM stylus as the ground truth

	1	2	3	4	5	6	7	8	Mean
Laparoscope	3.14	2.81	2.83	2.30	2.98	3.10	3.61	3.09	2.98
LUS	1.34	1.23	1.33	1.39	1.27	1.41	1.37	1.26	1.32

## 4 Discussion

In Experiment 1 (Section 2.A), we decided not to use the ring feature of the Hummel Plate for the evaluation of orientation measurement accuracy, as there is no ground truth for the axis of rotation in the tracker's coordinate system. This axis is the normal of the Hummel Plate which is essential for calculating the angle increment measured by the tracker to compare with the known increment of 11.25°. The original paper by Hummel et al.^13^ did not mention how this information was obtained in their study. For the NDI Tabletop Field Generator (TTFG) used in our study which has a large flat surface to enable securing on top the plywood platform supporting the Hummel Plate, we found that the variance of the z coordinates measured for each level is under 0.14 mm;^2^ hence, the z axis of the coordinate system could be considered orthogonal to the Hummel Plate and used as the axis of rotation. This is the assumption used in the work of Bonmati et al.^10^ For the optical trackers, it is not practical to align one of the axes with the normal of the Hummel Plate. While it would be possible to use a tracked pointer to determine the plane of the Hummel Plate, then compute the surface normal and use it as the rotation axis, we believe that such a method cannot serve as a ground truth as it is based on the position measurements of the tracker, which is the device being evaluated.

In the relative position error assessment using the Hummel Plate, we see that while optical trackers appear to have an intrinsically better tracking capability, as expected, their accuracy in measuring relative positions decreases significantly with longer distances (Table 4). Here, we give a possible explanation, by looking at Eq. (1). Suppose a point *P* at depth *z*_1_ has disparity *d*_1_, and when it moves to depth *z*_2_, the disparity becomes *d*_2_. Hence, the distance point *P* traveled will be $z_{2}-z_{1}=bf\left(\frac{1}{d_{2}}-\frac{1}{d_{1}}\right)$, assuming *z*_2_ *> z*_1_. Suppose there constructed depths are $z_{1}^{\prime }$ and $z_{2}^{\prime }$ with disparity measurement errors of $\Delta d_{1}$ and $\Delta d_{2}$, respectively. Then, we have the measured distance $z_{2}^{\prime }-z_{1}^{\prime }=bf\left(\frac{1}{d_{2}+\Delta d_{2}}-\frac{1}{d_{1}+\Delta d_{1}}\right)$. The disparity measurement error ∆*d* is a result of error in locating the markers in the left and right images (i.e., determining *x*_*l*_ and *x*_*r*_ in Fig. 2), which is due to factors such as image noise and distortion and the algorithm employed to match corresponding image points. It is often modeled to be normally distributed around the true two‐dimensional location of the markers.^32^ In practice, however, systematic errors due to environment or other factors often occur.^8^ If $\Delta d_{1}≃\Delta d_{2}$, that is, there is some systematic error in disparity measurement, the error in distance measurement ∆*s* can be put down as:

(5) $$\begin{array}{cc} \Delta s & =\left(z_{2}-z_{1}\right)-\left(z_{2}^{\prime }-z_{1}^{\prime }\right)\\ & ≃bf\left(d_{1}-d_{2}\right)\left[\frac{1}{d_{1}d_{2}}-\frac{1}{\left(d_{1}+\Delta d_{1}\right)\left(d_{2}+\Delta d_{2}\right)}\right] \end{array}$$

The above equation indicates that if both ∆*d*_1_ and ∆*d*_2_ are positive (negative), then ∆*s* is positive (negative), which means the measured distance is shorter (longer) than the actual distance, and the further distance the point moves (which leads to bigger *d*_1_ *− d*_2_), the bigger the error in the distance measured, which could explain our experiment results. Note that error in disparity measurement also affects the *x* and *y* coordinates determined, but most significantly the *z* coordinates. To further demonstrate this, let us look at the relative position error when the acetal block was positioned on the Hummel Plate in Direction 2, with the optical marker set facing straight at the optical cameras. We compute the relative position error when the block is moving along the rows and along the columns of the grid separately, and the results are shown in Table 9. When the block moves along the columns of the grid, it is roughly moving in the depth direction of the optical trackers, and we can see the error increases dramatically with longer distances, while the error increase is not significant when the block is moving along the rows of the grid, the direction approximately perpendicular to the depth direction of the optical trackers.

**Table 9:** RMS errors (in mm) of relative positions measured by Atracsys (optical), Spectra (optical), and Aurora (EM), with the acetal block moving on the Hummel Plate (a) along the rows and (b) along the columns, the column direction being approximately the depth direction of the optical cameras

	Atracsys	Spectra	Aurora
(a) RMS errors for distances along the rows			
50 mm	0.141	0.144	0.280
150 mm	0.161	0.167	0.419
250 mm	0.252	0.217	0.598
(b) RMS errors for distances along the columns			
50 mm	0.305	0.337	0.151
150 mm	0.945	1.044	0.276
250 mm	1.563	1.726	0.282

There is a possibility that a lever effect would be created, which is unfavorable to the optical trackers, if the acetal block was not placed level on the surface of the Hummel Plate. To inspect whether this had happened in Experiment 1, we fit a plane to the mean positions (i.e., position jitter is eliminated) of the 8 *× *6 grid points for each tracker and look at the RMS residual of the fitting. The underlining assumption is that if at some grid points, the acetal block was not placed level on the surface of the Hummel Plate, the mean positions from the measurements of the optical trackers would be “out‐of‐plane” at those points, and the RMS residuals of the plane fitting would be bigger than that for the EM tracker. The RMS residuals calculated for Level 3 are shown in Table 10. The results for Level 1 and Level 2 follow the same trend. Hence, we can consider that the acetal block was aligned with the surface of the Hummel Plate with accuracy during the experiment.

**Table 10:** RMS residuals (in mm) of plane fitting of the mean grid positions of Level 3 from the measurements of Atracsys (optical), Spectra (optical), and Aurora (EM) in two directions

	Atracsys	Spectra	Aurora
Direction 1	0.086	0.093	0.198
Direction 2	0.186	0.189	0.206

## 5 Conclusion

Our experiments have confirmed existing results in the literature that, in terms of tracking individual marker sets or sensors, optical trackers exhibit better tracking capability than EM trackers. However, we have found that, for optical trackers, the accuracy of measuring relative positions drops significantly with longer distances, due to decrease in tracking accuracy with increasing depth which is intrinsic to optical trackers, as well as possible systematic errors, whereas EM tracking is more consistent in this respect. In the case of a typical laparoscope, we used a stylus tip and demonstrated more precise and more accurate measurement of the stylus tip location with EM tracking than with optical tracking, as optical tracking is limited by the lever‐arm effect. For LUS probes, with an articulated tip, optical tracking is not appropriate and an embedded EM sensor is the natural choice. A system whereby both laparoscope and LUS are tracked via embedded EM sensors, as the one tested in this paper, would be a straightforward solution. We believe by integrating EM sensors into each device through careful design to place the sensor as close to the origin of the imaging coordinate system as possible to reduce the lever‐arm effect, EM tracking could provide more accurate image guidance than optical tracking. Our prototype of a combined, EM‐tracked laparoscope and LUS system, using representative calibration methods and assessed with an NDI EM stylus tool, showed a RMS localization error of 3.0 mm for the laparoscope and 1.3 mm for the LUS probe. The long‐term sustainability, manufacturing process, and eventual cost of such a system are factors to be considered by the vendor.

## Conflicts of interest

The authors have no conflicts to disclose.
